# Synthesis and FT-IR/Raman Characterization of a Graphene Oxide–Methacrylamide Monomer for Dental Applications

**DOI:** 10.3390/ma18153550

**Published:** 2025-07-29

**Authors:** Gennaro Ruggiero, Davide Di Rosa, Francesco Caso, Roberto Sorrentino, Fernando Zarone, Giuseppe Caso

**Affiliations:** 1 Scientific Unit of Digital Dentistry, Department of Neurosciences, Reproductive and Odontostomatological Sciences, Division of Prosthodontics, University “Federico II” of Naples, 80131 Naples, Italy; roberto.sorrentino@unina.it (R.S.); zarone@unina.it (F.Z.); 2 Department of Mathematics and Physics, University “Luigi Vanvitelli”, 81100 Caserta, Italy; giuseppe.caso@unicampania.it; 3 Department of Chemical Sciences, University “Federico II” of Naples, 80126 Naples, Italy; francesco.caso511@outlook.it

**Keywords:** GRAPHYMERE, graphene oxide, polymer, graphene, dental materials, composite

## Abstract

Background: Graphene oxide (GO) is widely explored as a functional additive in polymer composites; however, its simple physical dispersion in dental resins often leads to poor interfacial stability and limited long-term performance. Covalent functionalization may overcome these limitations by enabling chemical integration into the polymer matrix. This study presents the synthesis and FT-IR/Raman characterization of GRAPHYMERE^®^, a novel graphene oxide-based monomer obtained through exfoliation, amine functionalization with 1,6-hexanediamine, and transamidation with methyl methacrylate. Methods: A novel GO-based monomer, GRAPHYMERE^®^, was synthesized through a three-step process involving GO exfoliation, amine functionalization with 1,6-hexanediamine, and transamidation with methyl methacrylate to introduce polymerizable acrylic groups. The resulting product was characterized using FT-IR and Raman spectroscopy. Results: Spectroscopic analyses confirmed the presence of aliphatic chains and amine functionalities on the GO surface. Although some expected signals were overlapped, the data suggest successful surface modification and partial insertion of methacrylamide groups. The process is straightforward, uses low-toxicity reagents, and avoids complex reaction steps. Conclusions: GRAPHYMERE^®^ represents a chemically modified GO monomer potentially suitable for copolymerization within dental resin matrices. While its structural features support compatibility with radical polymerization systems, further studies are required to assess its mechanical performance and functional properties in dental resin applications.

## 1. Introduction

The field of polymeric resins is evolving rapidly, driving advancements across numerous technological and industrial sectors. Renowned for their versatility and adaptability, polymeric resins play a central role in applications spanning the biomedical [1], automotive [2], aerospace [3], bioengineering [4], and electronics industries [5]. The development of polymers with enhanced physical, chemical, and biological properties is crucial to addressing the diverse needs of these applications.

Enhancing polymeric materials through doping is a significant research area aimed at improving their performance characteristics. Doping involves introducing various atoms or molecules into the polymer matrix, resulting in properties such as increased conductivity, mechanical strength, and biocompatibility [6,7]. Among these doping agents, graphene oxide (GO) stands out as a particularly promising candidate. Derived from graphene, GO exhibits exceptional mechanical, electrical, and thermal properties, propelling it to the forefront of materials science research [8]. GO offers a pathway to advanced polymer composites with superior performances [9].

GO serves as an effective reinforcing agent for polymer composites, enhancing mechanical properties, improving barrier performance, and exhibiting antimicrobial activity. Its rigid, layered structure acts as a physical barrier against deformation, increasing hardness and resistance to indentation and scratching [10]. GO’s high modulus of elasticity enhances the stiffness of composite materials [11], while its ability to inhibit crack initiation and propagation reduces cyclic fatigue and enhances fracture toughness [12,13]. By bridging cracks and absorbing stress, GO prevents catastrophic failure, making the material more ductile and resistant to brittle fracture [14].

GO’s dense and layered structure also impedes the passage of molecules such as oxygen, moisture, and other gases through the polymer matrix [15]. This barrier property is vital for applications requiring protection against oxidation, moisture absorption, and degradation. Additionally, GO’s chemical inertness and robust structure contribute to the composite’s resistance to harsh chemical environments, preserving its integrity and performance over time [16]. GO effectively reduces the rate of oxidation, thereby extending the material’s functionality [17].

Beyond its mechanical and barrier properties, GO exhibits broad-spectrum antimicrobial activity against a wide range of bacterial and fungal pathogens [18]. It effectively targets medically significant pathogens such as gram-positive (e.g., Staphylococcus aureus, Enterococcus faecalis) and Gram-negative bacteria (e.g., Escherichia coli), and fungi (e.g., Candida albicans) [19,20,21]. GO’s ability to combat multidrug-resistant pathogens makes it a valuable tool in addressing the global challenge of antibiotic resistance [22]. GO disrupts bacterial membranes through electrostatic interactions and hydrophobic forces, leading to membrane distortion, increased permeability, and leakage of intracellular components, ultimately causing cell death [23,24]. In addition to disrupting cell membranes, GO impairs bacterial growth by targeting critical cellular processes [25]. Its large surface area and functional groups bind to essential nutrients, depriving bacterial cells of vital resources [25]. GO also interacts with key enzymes, disrupting metabolic pathways and further inhibiting bacterial proliferation [26]. These multifaceted antimicrobial mechanisms underscore GO’s potential in biomedical and dental applications.

In these fields, GO-doped composite materials are already demonstrating advantages, particularly in enhancing mechanical strength, barrier properties, and antimicrobial activity [27]. However, a simple physical dispersion of GO within polymer matrices has limitations in terms of stability and performance. Covalent bonding between GO and polymers represents an interesting advancement. Studies show that covalent integration ensures the uniform dispersion of GO throughout the polymer matrix, unlocking its full potential [28,29]. This stronger interfacial connection enhances mechanical properties, rendering the composite material tougher and more resilient. Additionally, covalent bonding improves barrier properties, increases resistance to gas and liquid permeation, and enhances biocompatibility for applications in drug delivery and biomedical devices [30].

Several functionalization strategies have been developed to covalently modify graphene oxide (GO), enhancing its applicability in various fields such as composites and biomedical materials. Key methods include reactions with diamines, diisocyanates, and silanes, as well as azide–alkyne click chemistry. These strategies aim to graft reactive functional groups onto GO, thereby enabling effective integration into polymer matrices through covalent bonding [31,32]. The involvement of different functional groups plays a crucial role in enhancing the compatibility and interaction between modified GO and the polymer phase, ultimately improving the mechanical and thermal properties of the composites [33,34].

While these methods have shown considerable effectiveness, many entail complex synthetic steps, the use of toxic reagents, or limited compatibility with dental monomers [35]. For example, a study highlighted that the intricate processes associated with traditional grafting approaches can hinder their practical application in real-world composites [36]. Notably, reactions employing photochemical methods or click chemistry are often regarded for their efficiency but may still require multi-step synthesis that can introduce drawbacks in terms of scalability and safety [37,38].

In light of the challenges posed by existing strategies, the present study proposes a simplified and sustainable approach to fabricate a polymerizable GO-based monomer via the utilization of 1,6-hexanediamine. This diamine has not been extensively described in the literature for such applications, indicating a novel pathway that could potentially sidestep the issues posed by the existing methods. By employing this diamine, the aim is to streamline the functionalization procedure while ensuring the resulting monomer maintains a high degree of reactivity and compatibility with dental and other polymeric systems [39,40]. This innovation could lay the groundwork for developing safer and more efficient biocompatible materials in dental applications.

The present research describes a sustainable and cost-effective method to create a versatile graphene oxide-based monomer, GRAPHYMERE^®^ (trademark registration number: 302024000066136). This monomer, achieved through the chemical modification of graphene oxide, can be combined with various dental and biomedical polymers.

This study introduces GRAPHYMERE^®^, a monomer designed to potentially form covalent bonds with polymer matrices through radical polymerization, a hypothesis to be confirmed by future studies. Unlike simple physical mixing, which often lacks long-term stability, covalent bonding ensures a robust interfacial connection. This strong anchoring prevents delamination and separation even under extreme conditions such as high temperatures, pressures, or mechanical stress [41]. The development of GRAPHYMERE^®^-based composites may represent a step forward in the design of advanced materials for dental applications.

## 2. Materials and Methods

### 2.1. Synthesis

**Figure 1:**
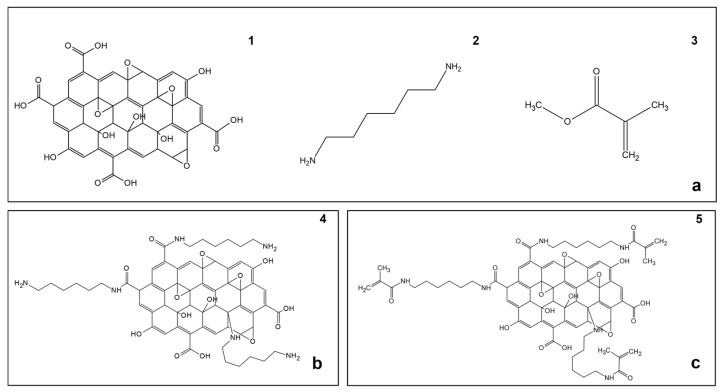
Reagents, intermediate product, and final product. (**a**) Molecular structure of the involved reagents: (**1**) Graphene oxide (GO), (**2**) 1,6-hexanediamine (ED), (**3**) methyl methacrylate (MMA); (**b**) Molecular structure of (**4**) graphene oxide functionalized with 1,6-hexanediamine (GO-ED); (**c**) (**5**) GRAPHYMERE^®^ supposed structure.

**Figure 2:**
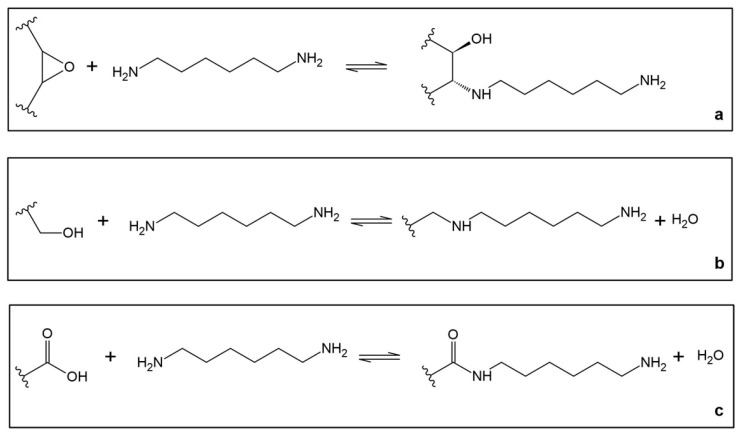
Functionalization of graphene oxide with 1,6-hexanediamine. The possible reaction for the insertion of 1,6-hexanediamine on GO is: (**a**) opening of an epoxide functional group; (**b**) nucleophilic substitution of a hydroxyl group; (**c**) transamidation of a carboxyl group.

**Figure 3:**
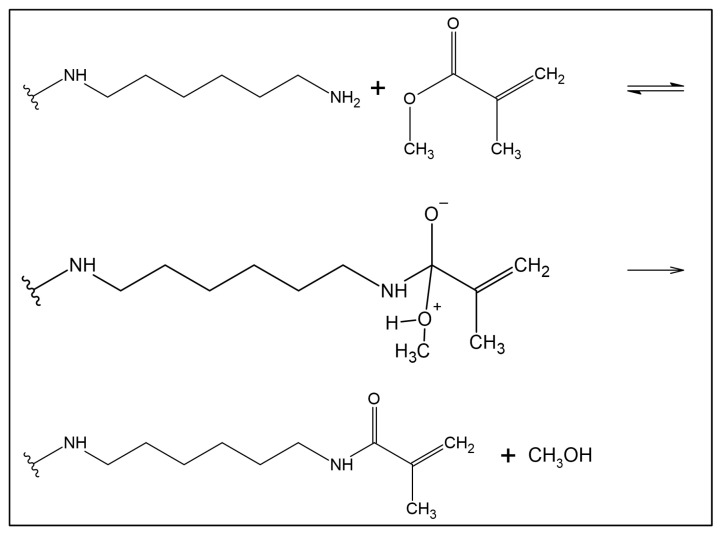
Acrylic functionalization: reaction of transamidation of methyl methacrylate with the amine group of 1,6-hexanediamine functionalized GO. It consists of a two steps reaction: a reversible (double arrows) nucleophilic attack of the amine functional group bound to the GO on the carboxyl of the MMA, followed by an irreversible (single arrow) elimination of a methanol molecule.

The synthesis of GRAPHYMERE^®^ is carried out using a three-step procedure (Figure 1): (1) exfoliation of GO; (2) amine functionalization of GO with 1,6-hexanediamine (ED) (Figure 2); (3) reaction of the exposed amine groups with methyl methacrylate (MMA) to convert them into acrylamide functional groups (Figure 3).

#### 2.1.1. Exfoliation

A dispersion of 280 mg of GO (Graphenea, San Sebastián, Spain, with 49–56% of carbon, 1–2% hydrogen, 0–1% nitrogen, 2–3% sulfur and 41–50% oxygen) was prepared in 130 mL of high-purity water (Milli-Q, Millipore^®^, Merck KGaA, Darmstadt, Germany), which was afterward sonicated in an ultrasonic bath for 30 min. The efficacy of the dispersion of GO using this method has already been shown by other authors [42].

#### 2.1.2. Functionalization with 1,6-Hexanediamine

The amination reaction (Figure 2) was carried out by adding 1.7 g of ED (>98% m/m, Thermo Fisher Scientific, Waltham, MA, USA) to the aqueous suspension of GO at room temperature for 4 h, keeping the system under stirring. The resulting graphene oxide functionalized with diamine (GO-ED, Figure 1) was separated from the aqueous phase by vacuum filtration on 0.45 μm mixed-cellulose ester (Millipore^®^, Merck KGaA, Darmstadt, Germany). The filtered product was first washed three times with Milli-Q water to remove the ED in excess, and then three times with acetone (Sigma-Aldrich^®^, 3050 Spruce Street, Saint Louis, MO 63103, USA, ACS reagent, ≥99.5%) to remove the water. The washed solid was then dried at room temperature. Previous research has already demonstrated the effectiveness of this procedure [43].

#### 2.1.3. Transamidation with Methyl Methacrylate

The dried product was resuspended in 30 mL of MMA (Lang Dental Manufacturing Company, Wheeling, IL, USA, solution-based methyl methacrylate >95% *m*/*v*, and N, N-dimethyl-p-toluidine <2% *m*/*v*) and put under stirring at ~60–70 °C for 24 h. In this reaction, MMA acts as both the solvent and reactant for the conversion of the exposed amine groups of GO-ED into acrylamide functional groups, as described in Figure 3. The resulting product, GRAPHYMERE^®^ (Figure 1), was filtered using vacuum filtration on a 0.45 μm filter mixed-cellulose ester, washed three times with acetone to remove the MMA in excess, and then dried at room temperature.

### 2.2. Characterization

The infrared and Raman spectra were obtained using, respectively, a Fourier Transformed Infrared spectrophotometer (FT-IR) (Thermo Nicolet 5700, Thermo OMNIC software 9.14, Thermo Fisher Scientific, Waltham, MA, USA), scanning the range 400–1800 cm^−1^, and a portable spectrophotometer Raman BRAVO (Bruker Optics, Billerica, MA, USA), equipped with a double laser source (DuoLaser™ Bruker, Billerica, MA, USA) and with SSE™ (Sequentially Shifted Excitation Bruker, Billerica, MA, USA) technology for the mitigation of fluorescence interferences, scanning the range 4000–400 cm^−1^.

## 3. Results

The final product is a fine black powder that cannot be dissolved in any solvent. It was characterized using solid-state Raman and FT-IR spectroscopy to obtain information about the chemical structure of the final product.

**Figure 4:**
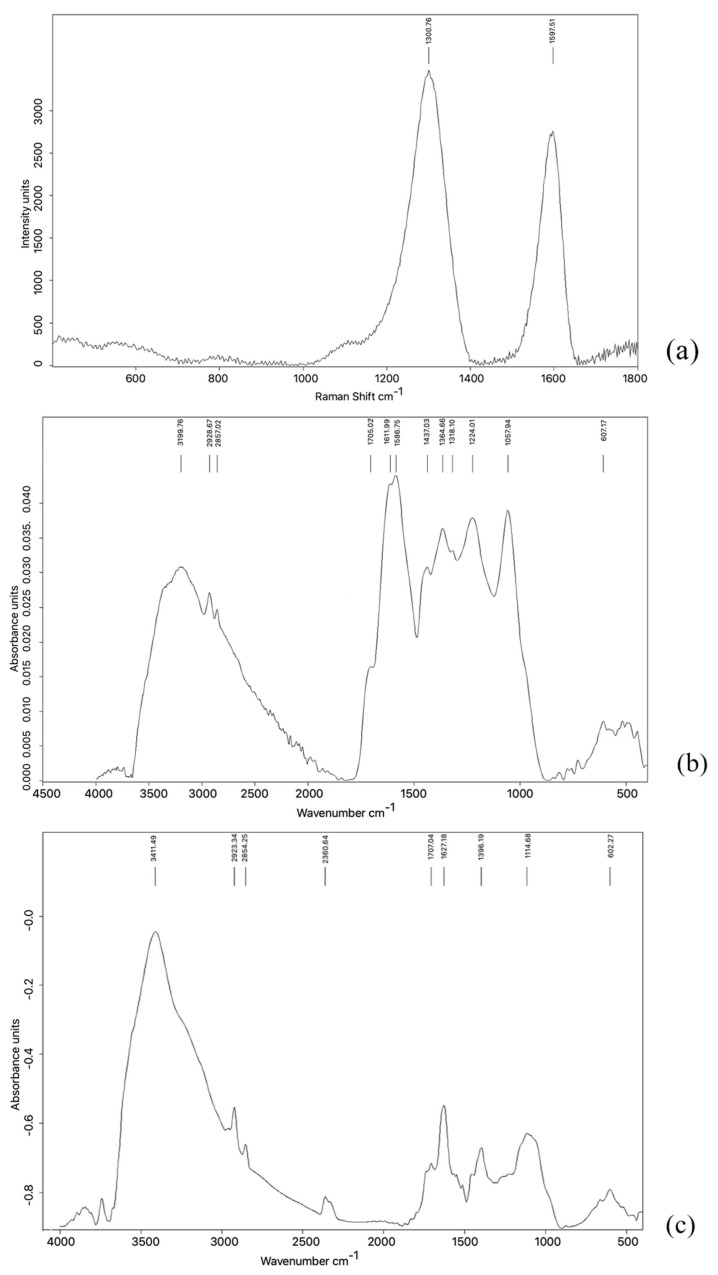
Raman and IR spectra of GRAPHYMERE^®^ and pristine GO. (**a**) The Raman spectrum of GRAPHYMERE^®^ shows two prominent bands at 1301 and 1598 cm^−1^, corresponding to the D and G bands typical of GO-based materials. (**b**) The IR spectrum of GRAPHYMERE^®^ displays absorption bands at 2929 and 2857 cm^−1^, assignable to aliphatic C–H stretching vibrations, and a broad band in the region 1550–1650 cm^−1^, consistent with N–H bending of amine groups. Bands related to GO functionalities (e.g., O–H and C=O stretching) may be overlapped by broader signals associated with methacrylamide moieties in the 3000–3400 cm^−1^ region. (**c**) The IR spectrum of pristine GO reveals intense and broad absorptions associated with hydroxyl (O–H, ~3200–3400 cm^−1^), carbonyl (C=O, ~1720 cm^−1^), epoxy (C–O–C, ~1220–1260 cm^−1^), and alkoxy (C–O, ~1050–1100 cm^−1^) groups.

The Raman and infrared spectra are shown in Figure 4.

The Raman spectrum of GRAPHYMERE (Figure 4a) shows two signals at around 1301 and 1598 cm^−1^ (D and G bands). The IR spectrum shows a large band in the range 3600 and 1800 cm^−1^, with peaks at 3200 cm^−1^, 2929 cm^−1^, and 2857 cm^−1^. In the range 1800–800 cm^−1^ there are many signals overlapping with each other; among these, there are evident peaks at: 1705 cm^−1^, 1611 cm^−1^, 1587 cm^−1^, 1437 cm^−1^, 1365 cm^−1^, 1318 cm^−1^, 1224 cm^−1^, and 1057 cm^−1^. Another low wide and low intensity band group can be found in the range 800–400 cm^−1^.

The IR spectrum of pristine GO (Figure 4c) exhibits a broad band in the 3600–2500 cm^−1^ region and several peaks between 1800 and 800 cm^−1^, with signals at approximately 1720, 1610, 1384, 1224, and 1047 cm^−1^. Additional low-intensity bands are visible below 800 cm^−1^.

## 4. Discussion

### 4.1. IR and Raman Spectra Interpretation

The two intense bands in the Raman spectrum (Figure 4a) (1301 and 1598 cm^−1^) are typically observed in carbon-based materials such as graphene oxide [44].

In pristine GO, a broad O–H stretching band (~3411 cm^−1^) dominates, attributed to hydroxyl groups and intercalated water.

In the GRAPHYMERE IR spectrum, this peak disappears, and the most plausible explanation is twofold: (1) Consumption or substitution of surface O–H groups: During the reaction, a fraction of the hydroxyl groups (responsible for the sharp maximum at 3411 cm^−1^) [45] is converted into amide bonds or into a less hydrogenated species, reducing the intensity of the free O–H stretching. (2) Modification of the hydrogen-bonding network and dehydration: Washing in organic solvents and the final drying steps remove most of the interlayer water, suppressing the sharp feature of “isolated” O–H groups. The residual O–H/N–H stretching appears as a weaker, broader band shifted to slightly lower wavenumbers (~3350–3200 cm^−1^) [45], merging into the baseline.

As for the –CH_2_ stretching bands observed at ~2929 and ~2857 cm^−1^ [45], it should be noted that they do not appear entirely de novo in the functionalized material (Figure 4c); faint traces of these bands were already visible in the pristine GO (Figure 4b). The marked post-reaction increase in their intensity is instead attributed to the higher number of aliphatic groups introduced through the hexyl linker bound to the acrylamide. Their enhanced visibility constitutes a strong indication of successful surface functionalization.

The peak at 1705 cm^−1^ remains nearly unchanged after the reaction, indicating that some surface carboxylic acid/ketone groups are not involved in the functionalization.

The expected Amide I (1690–1620 cm^−1^) and Amide II (~1550 cm^−1^) bands [45] are likely masked by the strong overlapping C=C and C=O signals characteristic of oxidized GO, as previously noted. This makes direct FT-IR confirmation of the acrylamide insertion particularly difficult.

The persistence of the 1356/1318 cm^−1^ (C–O/C–N) and 1058 cm^−1^ bands suggests that most epoxide and phenolic groups remain on the surface, in agreement with a partial functionalization [45].

The increased intensity at 1437 cm^−1^ is consistent with δ(CH_2_) vibrations of the hexyl chains [45]. Nonetheless, a possible contribution from residual water should be considered.

In the fingerprint region (<1000 cm^−1^), both materials show a similar spectral profile. Minor low-intensity differences in this region do not offer conclusive information regarding new functional groups.

These FT-IR data thus only partially support the hypothesis of a successful functionalization of GO with acrylamide via a hexyl linker. However, the actual realization of this reaction is supported by data in the literature [46].

### 4.2. Co-Polymerization of Suitable Molecules

GRAPHYMERE^®^ has acrylic functional groups that have been inserted into graphene oxide to make it compatible for radical co-polymerization with the most used resins in the field of dentistry. Many of these resins come from acrylic precursors, so they are compatible for polymerization with GRAPHYMERE^®^.

**Figure 5:**
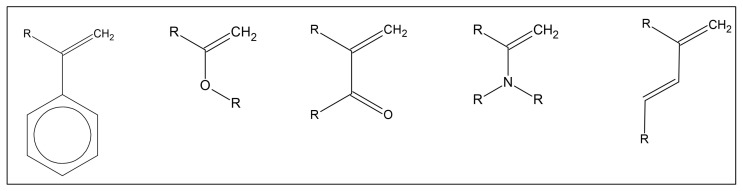
Examples of unsaturated functional groups capable of copolymerization with GRAPHYMERE^®^. All molecules that have unsaturated bonds can copolymerize with GRAPHYMERE^®^ through a radical mechanism.

In general, all monomers of any kind, which are capable of polymerizing via radical mechanisms, are potentially able to be chemically bound with GRAPHYMERE^®^ [47]. These types of molecules usually present an unsaturated double C=C bond, which is often conjugated with another functional group that can stabilize their radical form (Figure 5) [48].

Some commonly used molecules in the biomedical field have all the characteristics to be considered suitable for copolymerization with GRAPHYMERE^®^, including, for example, methyl methacrylate, styrene, vinyl chloride, and BIS-GMA (Bisphenol A-glycidyl methacrylate) [49].

On average, since every sheet of GO has more than one function that can be functionalized with hexamethylenediamine, every GRAPHYMERE^®^ sheet can have more than one N-methyl acrylamide function; this implies that GRAPHYMERE^®^ introduces a variable degree of cross-linking in the polymers bound to it, depending on its concentration [48,50]. Of course, if it is added to resins, which already have a high degree of cross-linking, it does not affect the properties of the polymer in this sense.

It has been shown that the simple dispersion of graphene oxide in polymers already shows enhancements to the physical properties of the doped materials. The chemical integration in the resin matrix of GRAPHYMERE^®^ as a co-monomer instead of a simple filler/additive reduces the risk of losing the embedded graphene oxide in the outer part of the material, like leaking due to polymer swelling caused by solvents (including water and human saliva) [50,51].

### 4.3. Comparison with Similar Materials

Several experimental strategies have demonstrated that the chemical modification of graphene oxide (GO) or related graphene materials can significantly enhance the mechanical, thermal, and electrical properties of polymer nanocomposites. For example, Ha et al. reported that chemically modified GO incorporated into a polyimide (PI) matrix resulted in remarkable improvements in tensile strength and modulus, up to 12–18 times higher than neat PI, due to an excellent dispersion and stress transfer efficiency [52]. Similarly, Park and Kim prepared epoxy nanocomposites using amine-functionalized graphene, achieving improved stiffness and electrical conductivity owing to strong interfacial bonding [53]. In a related study, Shagor et al. employed silanized GO in carbon fiber-reinforced composites, observing enhancements in both mechanical and thermal properties, confirming the efficiency of silane-based functionalization [54]. Zhang et al. also demonstrated that GO functionalization can optimize the interfacial microstructure in carbon fiber composites, resulting in increased adhesion and overall composite performance [55]. Furthermore, Omelchenko et al. developed thin nanocomposite layers based on complexes of polyaniline and GO, highlighting how non-covalent interactions between conductive polymers and GO can improve surface morphology and electrical behavior [56].

In addition, other studies have focused on covalent approaches to functionalizing GO for nanocomposite fabrication. Yan et al. [43] reported the preparation of amino-functionalized GO for use in PMMA-based nanocomposites, showing improved mechanical and thermal properties, although the covalent nature of the linkage limited its compatibility across different polymer matrices. Ferreira et al. highlighted how GO functionalized with epoxy-compatible groups improved reinforcement effects in epoxy nanocomposites, particularly due to better dispersion and interfacial adhesion [57]. Chee et al. and Thickett & Zetterlund provided comprehensive reviews of various polymer/graphene nanocomposite systems, focusing on the structure–property relationships influenced by GO functionalization and the advantages and drawbacks of different chemical grafting strategies [58,59].

Compared to these approaches, which typically rely on pre-functionalization steps using diamines, isocyanates, or silane agents, the functionalization strategy proposed in this work offers significant advantages. The use of GRAPHYMERE^®^ dispersed in an acrylic/olefinic solution, followed by in situ polymerization, allows for homogeneous GO dispersion, a broad polymer compatibility, and the avoidance of toxic reagents and solvents. As a result, the resulting nanocomposites exhibit enhanced interfacial adhesion, mechanical robustness, and thermal stability, while offering a more sustainable and cost-effective alternative to conventional covalent functionalization routes. The previous discussion is briefly summarized in Table 1.

### 4.4. Relevance and Potential of GRAPHYMERE^®^ in Dental Applications

Although the present work is focused on the synthesis and preliminary chemical characterization of GRAPHYMERE^®^ (GO-ED-MA), we believe it is appropriate to briefly elaborate on its potential relevance in the dental field.

GRAPHYMERE^®^ has been designed to covalently integrate with acrylic resins through radical polymerization, a property that aligns closely with the chemistry of many commonly used dental materials. For this reason, we consider it reasonable to anticipate its applicability in various areas of restorative and prosthetic dentistry. In particular, its structure and functional reactivity make it potentially compatible with resins used for fabricating prosthetic bases, artificial teeth, relining and repairing materials, as well as glazing agents and frameworks. Furthermore, its use could be extended to orthodontic and gnathological appliances such as retainers, splints, bite guards, and sleep apnea devices. Other promising uses might include enamel–dentin adhesives, cements, endodontic posts, custom trays, surgical templates, and temporary reconstructions.

The integration of GRAPHYMERE^®^ into such materials could ultimately contribute to treating a wide range of dental and maxillofacial conditions, including edentulism, carious lesions, dental fractures, bruxism, malocclusions, and anatomical defects.

These potential uses, however, remain theoretical. While its chemical structure supports compatibility with dental resins, further mechanical, biological, and clinical studies are necessary to validate its performance and safety.

### 4.5. Limitations

This study is primarily focused on the synthesis and characterization of GRAPHYMERE^®^, introducing its potential applications and compatibility with various polymers and monomers. While the results highlight promising features of the material, such as its ability to integrate into polymer matrices through covalent bonding, the scope of this work does not extend to an in-depth evaluation of its mechanical, chemical, or biological properties.

The material’s potential advantages in biomedical and dental applications, as well as its compatibility with other molecules, remain theoretical until validated through targeted scientific investigations. Comprehensive studies are required to assess GRAPHYMERE^®^’s performance in terms of specific mechanical strengths, chemical stability, and biological behavior under practical conditions.

A key limitation of this study is the absence of direct proof confirming the proposed reaction steps. Despite attempts with both solution and solid-state NMR, the material’s low solubility and structural complexity prevented a clear spectral interpretation.

A further limitation concerns the potential attenuation of some biological activities of GO as a consequence of its chemical functionalization. While such modifications are often pursued to enhance biocompatibility or enable specific applications, they may also suppress beneficial antimicrobial or cytotoxic effects originally associated with native GO. This trade-off should be carefully considered in the design of functionalized materials, especially when biological activity is a desired feature [60].

Future research will be crucial to determining its real-world applicability, ensuring that the proposed benefits align with the demands of its intended applications. This study is intended as a foundational step, providing a starting point for further experimental and applied research.

## 5. Conclusions

This study presents the synthesis and FT-IR/Raman characterization of GRAPHYMERE^®^, a novel graphene oxide-based monomer obtained through a three-step process involving 1,6-hexanediamine. Its synthesis is economically sustainable, environmentally friendly, and results in a material designed to be potentially compatible with dental polymers through radical polymerization mechanisms.

GRAPHYMERE^®^ may serve as a platform capable of copolymerizing with dental polymers via radical mechanisms. Future studies are mandatory to validate the hypothesized benefits through targeted mechanical, chemical, and biological testing aligned with specific applications in the dental field.

## 6. Patents

The chemical structure of GRAPHYMERE^®^ and the above-described synthesis procedure were deposited at the Italian Patent and Trademark Office (application number 202024000001687) on 17 April 2024.

## Figures and Tables

**Table 1:** Overview of GO functionalization strategies and related polymer nanocomposites from selected literature, including the present work.

Article	Synthesis Method	Polymer Compatibility	Applications	Advantages	Limitations
GO-ED-PMMA (Yan et al., 2012) [43]	Amine functionalization + in situ PMMA polymerization	PMMA	Optical/electronic materials	Improved adhesion	Limited polymer scope
GO-epoxy (Ferreira et al., 2018) [57]	GO functionalized with epoxy-reactive agents	Epoxy resins	Structural/engineering materials	Enhanced mechanical and thermal properties	Poor compatibility with flexible or biocompatible systems
GO/polymer review (Chee et al., 2015) [58]	Dispersion, grafting, and in situ polymerization	Broad	General-purpose nanocomposites	Comprehensive overview of methods	Scalability and reproducibility issues
GO-acrylic (Thickett & Zetterlund, 2013) [59]	Covalent functionalization on –OH/–COOH/epoxide groups	Acrylics, colloids, and hydrogels	Sensors, coatings, and advanced nanomaterials	High reactivity and tunability	Complex reagents or harsh conditions
GRAPHYMERE^®^ (this work)	Exfoliation, amination, and acryl functionalization	Acrylics, vinyls, and BIS-GMA	Dental and biomedical	Uniform dispersion + low-toxicity solvents	Functional properties are still under investigation
Polyimide/GO (Ha et al., 2012) [52]	Carbodiimide activation + polyimide polymerization	Polyimides	High-temperature electronics and aerospace	High-performance (mechanical/electrical/thermal)	Uses less benign reagents
PANI/GO films (Omelchenko et al., 2014) [56]	In situ aniline polymerization	Polyaniline	Conductive coatings and sensors	Improved conductivity and thermal stability	Requires strong acids/oxidizers
Epoxy/amineGO (Park & Kim, 2014) [53]	Amine-functionalized GO + epoxy curing	Epoxy resins	Structural composites and coatings	Uniform dispersion and impact resistance	Requires organic solvents and coupling agents
CF w/silanized GO (Shagor et al., 2020) [54]	Silanization of GO + carbon fiber composite fabrication	Carbon fiber/epoxy	Aerospace and automotive composites	Enhanced mechanical and thermal stability	Moderate cost and irritant silanes
CF/GO interphase (Zhang et al., 2012) [55]	GO coating on carbon fiber + epoxy impregnation	Carbon fiber/epoxy	High-strength composites	Fracture-resistant interphase	Relies on conventional epoxy resins

## Data Availability

The data presented in this study are available on request from the corresponding author due to patents related to the data.

## Abbreviations

The following abbreviations are used in this manuscript:

GO	Graphene Oxide
ED	1,6-hexanediamine
MMA	methyl methacrylate
FT-IR	Fourier Transformed Infrared
